# The many meanings of Alzheimer’s disease and why they matter for policy, research, and care

**DOI:** 10.1007/s11357-026-02131-z

**Published:** 2026-02-09

**Authors:** Roderick A. Corriveau

**Affiliations:** 1 Department of Neurology, Northwestern University Feinberg School of Medicine, Chicago, USA; 2 Circle BioPharma, Pittsburgh, USA

**Keywords:** Alzheimer's disease, Biomarkers, Genetics, Pathology, Dementia, Health Policy

## Abstract

Alzheimer’s disease (AD) is a national priority with far-reaching implications for patients, families, clinicians, researchers, and policymakers. Yet the term AD refers to multiple distinct meanings that are often overlooked or ambiguously applied, risking misaligned research and policy priorities that affect patient outcomes. Recent FDA approvals of passive immunotherapies and related biomarker developments show that differing interpretations of AD complicate research, regulatory decisions, payer coverage, and clinical use. In contrast to efforts aimed at revising AD nomenclature, this paper clarifies five key meanings of AD as currently used. It provides a practical framework to indicate how the term AD is applied in different ways within and across clinical, research, regulatory, and policy contexts. Each key meaning—clinical (symptom-focused), genetically determined (mutation-driven), pathological (autopsy-confirmed), plaque-defined (amyloid-beta–plaque-positive), and broad (can encompass multiple dementia types)—may apply alone or in combination depending on context. These distinctions also raise ethical considerations, particularly for biomarker-based diagnoses and their impact on patient communication and decision-making. Using this framework helps decision-makers identify the intended AD meaning and align research, policy, and care for better patient outcomes.

What is Alzheimer’s disease? Over years of working with leading clinicians and researchers, I have encountered differing answers: some emphasize cognitive decline or use AD interchangeably with dementia; others point to genetically determined forms; still others focus on hallmark protein changes in the brain. These divergent uses reflect multiple meanings of AD [1], which may be applied alone or in combination, even by the same person in a single conversation. They influence FDA approvals, Medicare coverage, trial eligibility, research priorities, and how clinicians communicate with patients and families. Table 1 provides a practical framework for navigating this complex landscape.

**Table 1:** Key meanings of Alzheimer’s disease in professional and everyday use

Term (alternative terms)	What it means	Defining features	Notes
**Clinical AD** (AD; AD dementia; sporadic AD)	Diagnosis based on dementia symptoms and clinical evaluation	AD-type dementia; AD pathology is not required	Most diagnoses are after age 60–65; common in routine clinical practice
**Genetically determined AD **(AD; autosomal dominant AD; genetic AD)	Diagnosis caused by rare, inherited autosomal-dominant mutations	A known causal mutation	Symptoms usually begin before age 60; rare; detectable by genetic testing
**Pathological AD** (AD; pathologic AD; AD neuropathologic changes; Sporadic AD)	Diagnosis defined by hallmark brain changes at autopsy	AD pathology; dementia not required	Has served as a reference standard
**Plaque-defined AD **(AD; sporadic AD)	Diagnosis proposed based on biomarkers in living individuals	Amyloid-β plaque biomarker positivity; dementia and tau tangles not required	Primarily research use; utility in asymptomatic people is debated
**AD as a broad category** (dementia, when used as a broad category)	Not a diagnosis; umbrella term for AD and may include other dementias	Varies by speaker; may include both AD and non-AD dementias	Used publicly and in advocacy/policy; interpretation varies

Two examples illustrate how misreading or conflating meanings of AD can misdirect research, complicate treatment development, pose difficulties for regulators, and impact patients and families. First, claims that AD is the leading cause of dementia imply more certainty than the evidence supports. AD is the most frequently diagnosed form, yet its underlying causes remain unclear in most cases [2]. Second, most people hear “AD” and assume dementia, even though in the U.S.A. it can be diagnosed solely based on brain protein changes [1]. Once observable only postmortem, these changes are now detectable in living individuals [2]. However, they do not necessarily predict cognitive decline or future dementia [1–6].

Because of variability in AD meanings and the ambiguity this can create for individual patient needs, recognizing the intended AD meaning is essential. Clinician-patient communication is more precise and effective when AD meanings are specified, helping patients understand their diagnosis and plan care, including appropriate monitoring. It also helps guide policymaker and payer decisions, including coverage criteria for therapies based on clinical, biomarker-defined, or genetically determined AD (or combinations), and aligns study inclusion criteria with the AD meaning used.

## FDA approvals and regulatory complexity in Alzheimer’s disease

The FDA’s 2021 accelerated approval of aducanumab (a monoclonal antibody treatment for AD) [7] demonstrates how differing interpretations of AD drive real-world evidentiary and regulatory complexities. A congressional report concluded that the decision relied on the hypothesized link between amyloid-β reduction and clinical benefit [8]. Although aducanumab lowers amyloid-β, evidence for meaningful clinical benefit remained limited and debated [9, 10]. The drug was withdrawn from the market in 2024 [11]. Prior to aducanumab’s approval, the FDA had not considered amyloid-β a valid pharmacodynamic biomarker for trials [8]. Two similar drugs soon followed, lecanemab [12] and donanemab [13], which may modestly slow cognitive decline but do not halt it and carry notable risks [14]. Approving these drugs amid unresolved evidence creates uncertainty for regulators, clinicians, patients, and payers [15, 16]. It underscores the urgent need to clarify the intended meaning of AD, from regulatory contexts to everyday care settings.

## Decoding five key meanings of Alzheimer’s disease

These cases highlight the challenge of turning mechanistic hypotheses into effective therapies when AD itself can be interpreted in multiple, often conflicting ways [1, 5]. Understanding these distinctions is essential for clear communication and sound policy. Drawing on experience with clinicians and other stakeholders, Table 1 outlines five key AD meanings applied across diverse professional and public contexts, each with distinct implications. Serving as a quick reference, this framework fosters shared understanding and helps align clinical, research, regulatory, and policy actions with the intended meaning of AD.

Key concepts defined for non-specialists: amyloid-β plaques (“plaques,” protein deposits in the brain); tau tangles (“tangles,” twisted protein fibers inside brain cells); AD pathology (plaques plus tangles); and biomarker (indicators of amyloid-β or tau). Because AD terminology varies widely, a selection of common alternative terms for each AD meaning is provided.

A few notable conclusions emerge from this overview:

- Several AD terms span multiple meanings: Alzheimer’s disease, Alzheimer’s, and AD; sporadic AD can apply to clinical, pathological, and plaque-defined AD.
- Some AD terms are meaning-specific: for example, autosomal dominant AD is used only in a specific diagnostic context, reflecting a rare inherited genetic cause.
- Defining features vary: however, individuals may simultaneously meet criteria for more than one AD diagnosis, reflecting overlap in observed characteristics.

The framework aligns with disease stages [17]. Individuals without symptoms but with biomarker evidence of future AD dementia risk, or those carrying gene mutations that cause AD, may be classified as preclinical AD. Those with mild cognitive decline and suspected progression to AD dementia may be diagnosed with mild cognitive impairment (MCI) or early-stage AD.

Although each of these five meanings serves a purpose, their overlap creates a complex landscape that directly affects patient care, research priorities, and regulatory and coverage decisions. They also reflect a core geroscience principle: late-life pathological and clinical syndromes frequently arise from the interaction of multiple biological processes.

Cognitive decline and dementia largely reflect this interplay, as multiple age-related influences on risk and resilience can shape outcomes in ways that remain incompletely understood. These include proteinopathies such as AD pathology, vascular dysfunction, chronic inflammation, metabolic dysregulation, and other co-occurring diseases and risk factors that vary in combination within individuals. Consequently, people with similar burdens of amyloid-beta plaques or tau tangles may experience markedly different cognitive trajectories, pointing to the limits of single-pathology definitions in complex late-life syndromes such as AD. The following sections examine each meaning and highlight opportunities to clarify AD’s scope and strengthen informed action in care, policy, and scientific inquiry.

## Clinical Alzheimer’s disease: the default basis for diagnosis, reimbursement, and care planning

Clinical Alzheimer’s disease [18–20], the most common and familiar form, typically emerges after age 60–65 (Table 1). It is characterized by dementia, including progressive declines in memory, thinking, and daily functioning. Diagnosis generally relies on cognitive testing, clinical evaluation, and patient history; additional tests may rule out other diagnoses. Apolipoprotein E (APOE) ε4, a gene variant associated with a higher incidence of clinical AD, is sometimes used to assess an individual’s risk. However, carrying this variant alone does not determine whether the person will develop clinical AD [21].

Diagnostic practices vary. Exclusion of other dementias often depends on the availability of specialized evaluations. Although amyloid-β plaques and tau tangles are hallmark features, they are not required for this diagnosis. Most with clinical AD also show additional dementia-related brain changes at autopsy, commonly described as mixed dementia, underscoring the molecular complexity clinicians must navigate [4].

Diagnostic and biochemical heterogeneity of clinical AD complicates treatment development and implementation. Insurance coverage may be affected; for example, recent drug approvals may benefit some patients while others face higher risks of side effects [22]. Aligning treatments with each patient’s clinical and molecular profile enables more effective, personalized care. It also underscores the need for AD meaning-specific guidance in coverage and policy.

## Genetically determined Alzheimer’s disease: a form with a confirmed cause

Most AD cases have no clearly identified cause, but rare autosomal dominant mutations in APP, PSEN1, and PSEN2 are causal, leading to dementia with amyloid-β plaques and tau tangles [23, 24]. For carriers, dementia is effectively inevitable, typically appearing in the 30s or 40s and almost always before age 60. About half of an affected individual’s children inherit the mutation. Those who inherit usually develop dementia at a similar age. “Familial AD” applies to these families but also refers to families with higher-than-typical incidence lacking a causal mutation.

AD in Down syndrome, caused by a trisomy, is also highly penetrant, though the age of onset and clinical course can vary [25]. By contrast, APOE ε4 elevates risk but is not determinative.

Genetically determined AD is often included under the broader heading of Alzheimer’s disease in policy and medicine. The National Alzheimer’s Project Act (NAPA) Reauthorization Act, for instance, shapes the national strategy [26, 27]. While this inclusive approach captures the spectrum of dementia, it can obscure critical distinctions. These include age of onset, progression, genetic counseling needs, and research or treatment priorities. Recognizing these differences guides clinical care, informs family planning, and focuses research and policy efforts more effectively.

## Pathological and plaque-defined Alzheimer’s disease: implications of biomarkers

Pathological AD has largely shaped the field’s thinking about how dementia develops across all forms of AD [1, 28]. It is identified by amyloid-β plaques and tau tangles in the brain (Table 1). Historically, this diagnosis was made only postmortem, which is still considered the reference standard. Notably, no universal standard for pathological or biomarker-based diagnosis of AD requires cognitive decline.

Recent advances, including FDA-approved, cleared, or authorized imaging and fluid-based biomarkers, allow the detection of these pathological changes in living individuals. However, detecting both plaques and tangles in living people remains uncommon outside research due to practical limitations [2, 3, 29, 30]. Ongoing research aims to complement amyloid-based biomarkers by integrating biomarkers for tau-related processes, other vascular and neurodegeneration pathways, and multimodal risk profiling.

These biomarker advances are closely tied to the amyloid hypothesis, which proposes that amyloid-β accumulation triggers a cascade leading to cognitive decline [31]. In drug development, the form of AD that is targeted and the desired outcomes shape trial eligibility, FDA approvals, and coverage decisions. In this context, some investigators and advocates have proposed defining a form of AD in living individuals solely on the presence of amyloid-β plaques (“plaque-defined AD”) [32]. This definition would not require evidence of tau tangles or symptoms. Adopting this approach could expand early detection and access to interventions.

Yet evidence suggests that about 20% of individuals with pathological AD never develop dementia, emphasizing the uncertainty of predicting outcomes from amyloid-β alone [4]. The proportion is higher when amyloid-β deposits occur without other brain pathology [33]. Targeting amyloid-β in asymptomatic individuals involves uncertain clinical benefit, ethical considerations, and regulatory issues [3].

Beyond treatment, assigning a plaque-defined AD diagnosis raises additional ethical, clinical, and regulatory questions. Individuals may experience distress upon learning they have amyloid-β pathology, even if asymptomatic. For clinicians and regulators, this diagnosis has major implications for patient care, trial eligibility, insurance coverage, and drug approvals. Managing these implications requires coordinated regulatory and reimbursement governance and a shared understanding of benefits, risks, and uncertainties across stakeholders [3, 6].

## Alzheimer’s disease as a broad category

AD is often used broadly, encompassing all AD forms and, at times, non-AD dementias, including the AD-related dementias defined in U.S. federal policy: frontotemporal dementias, Lewy body dementias, vascular contributions to cognitive impairment and dementia (VCID), and mixed dementias [26, 27, 34]. This usage is common in advocacy, awareness campaigns, policy discussions, and everyday conversation. It simplifies messaging by highlighting the profound impact on patients, families, and caregivers.

However, growing evidence indicates that mixed brain pathologies are the rule rather than the exception in older adults, including those diagnosed with AD [4]. The framework in Table 1 may help reduce the marginalization of mixed dementias by highlighting the heterogeneity and complexity of AD cases, which typically include mixed pathologies. This also informs consideration of prevention and intervention strategies targeting additional pathways, including non-AD proteinopathies and potentially modifiable risk factors such as VCID.

At the same time, using AD as a broad term can obscure critical distinctions across clinical, research, and policy contexts. To communicate clearly and avoid misunderstandings, policymakers and payers should specify the intended meaning. Substantial clinical and molecular differences exist across and within AD meanings; blurring these distinctions can undermine patient care, complicate scientific priorities, and contribute to policy that does not account for the multiple forms of AD. Because AD is often used broadly in high-level discussions, timely clarification for policymakers and stakeholders at critical junctures ensures informed, actionable decisions across the AD ecosystem.

## Conclusion: toward clearer communication for effective decision-making

AD encompasses clinical, genetic, pathological, and biomarker-defined forms. These may occur alone or in combination, influencing diagnosis, care, research, regulation, and public understanding. While efforts to develop consensus terminology [35] aim to establish formal nomenclature, Table 1 provides a practical framework that clarifies how AD is used today and strengthens coordinated action across care, regulation, and research.

Policymakers, clinicians, researchers, and advocates should explicitly specify the intended AD meaning in professional communications. Doing so ensures consistent regulation, evidence-based coverage, ethical alignment, coordinated bench-to-bedside research, and transparency. Specifying “AD” is more than semantics: it protects patients, guides research, and anchors policy in clinical and molecular realities.

Transparent communication about AD is also essential to ensure that patients and families receive care grounded in a personalized understanding of what an AD diagnosis means for them, along with informed consideration of available treatments. It may also enhance prevention strategies by encouraging interventions targeting multiple and modifiable risk pathways that arise during aging, rather than focusing solely on single molecular targets.

## Data Availability

Not applicable (commentary, no original data).
